# The genetic heritage of Alpine local cattle breeds using genomic SNP data

**DOI:** 10.1186/s12711-020-00559-1

**Published:** 2020-07-14

**Authors:** Gabriele Senczuk, Salvatore Mastrangelo, Elena Ciani, Luca Battaglini, Filippo Cendron, Roberta Ciampolini, Paola Crepaldi, Roberto Mantovani, Graziella Bongioni, Giulio Pagnacco, Baldassare Portolano, Attilio Rossoni, Fabio Pilla, Martino Cassandro

**Affiliations:** 1 Dipartimento di Agricoltura, Ambiente e Alimenti, University of Molise, 86100 Campobasso, Italy; 2 Dipartimento Scienze Agrarie, Alimentari e Forestali, University of Palermo, 90128 Palermo, Italy; 3 Dipartimento di Bioscienze Biotecnologie e Biofarmaceutica, University of Bari, 70124 Bari, Italy; 4 Dipartimento di Scienze Agrarie Forestali e Alimentari, University of Torino, 10095 Grugliasco, Italy; 5 Dipartimento di Agronomia Animali Alimenti Risorse naturali e Ambiente, University of Padova, 35020 Legnaro, Italy; 6 Dipartimento di Scienze Veterinarie, University of Pisa, 56100 Pisa, Italy; 7 Dipartimento di Scienze Agrarie ed Ambientali - Produzione, Territorio, Agroenergia, University of Milano, 20133 Milan, Italy; 8 Istituto Sperimentale Italiano Lazzaro Spallanzani, Loc. La Quercia, 26027 Rivolta d’Adda, CR Italy; 9 Istituto di Biologia e Biotecnologia Agraria (IBBA), CNR, 20133 Milan, Italy; 10 Italian Brown Cattle Breeders’ Association, Loc. Ferlina 204, 37012 Bussolengo, VR Italy

## Abstract

**Background:**

Assessment of genetic diversity and population structure provides important control metrics to avoid genetic erosion, inbreeding depression and crossbreeding between exotic and locally-adapted cattle breeds since these events can have deleterious consequences and eventually lead to extinction. Historically, the Alpine Arc represents an important pocket of cattle biodiversity with a large number of autochthonous breeds that provide a fundamental source of income for the entire regional economy. By using genotype data from medium-density single nucleotide polymorphism (SNP) arrays, we performed a genome-wide comparative study of 23 cattle populations from the Alpine Arc and three cosmopolitan breeds.

**Results:**

After filtering, we obtained a final genotyping dataset consisting of 30,176 SNPs for 711 individuals. The local breeds showed high or intermediate values of genetic diversity compared to the highly selected cosmopolitan breeds. Patterns of genetic differentiation, multidimensional scaling, admixture analysis and the constructed phylogenetic tree showed convergence, which indicates the presence of gene flow among the breeds according to both geographic origin and historical background. Among the most differentiated breeds, we identified the modern Brown cattle. In spite of admixture events, several local breeds have preserved distinctive characteristics, which is probably due to differences in genetic origin and geographic location.

**Conclusions:**

This study represents one of the most comprehensive genome-wide analysis of the Alpine cattle breeds to date. Using such a large dataset that includes the majority of the local breeds found in this region, allowed us to expand knowledge on the evaluation and status of Alpine cattle biodiversity. Our results indicate that although many of the analyzed local breeds are listed as endangered, they still harbor a large amount of genetic diversity, even when compared to some cosmopolitan breeds. This finding, together with the reconstruction of the phylogeny and the relationships between these Alpine Arc cattle breeds, provide crucial insights not only into the improvement of genetic stocks but also into the implementation of future conservation strategies.

## Background

Assessment of genetic diversity and population structure is a fundamental task, not only to understand the evolutionary history of the origin of breeds, but also to provide important information for the conservation and management of local biodiversity [1, 2]. Indeed, evaluating the genetic diversity within and between populations provides an important control metric to avoid genetic erosion, inbreeding depression and cross-breeding between exotic and locally-adapted cattle breeds that can have deleterious consequences and eventually lead to extinctions [3–5]. Among livestock species, *Bos taurus* is considered as having the largest number of breeds at risk of extinction, which results in gradual depletion of their genetic diversity [6]. Indeed, over the last 15 years, extinction of many local breeds has been reported by the FAO [7]. In Europe, half of the breeds that existed at the beginning of the twentieth century have become extinct and a third of the remaining 770 breeds are in danger of disappearing during the next 20 years [8]. Unplanned genetic introgression and crossbreeding have largely contributed to the loss of indigenous breeds. Within this context, increasing the knowledge on the genomic architecture of these breeds is crucial for both maintaining and improving proper selection strategies. Moreover, actions that take the ever-changing environmental conditions and market demands into account are becoming increasingly essential.

Historically, the Alpine Arc represents an important pocket of cattle biodiversity with its large number of autochthonous breeds. These breeds provide a fundamental source of income for the entire regional economy, since they are fully adapted to the difficult mountain environment and carry important traits for livestock production [9]. It is not by chance that, during the last century, some of the bovine breeds from the Alpine Arc have been used for genetic improvement and have spread over different parts of the world. As an example, a few individuals from the original Braunvieh that originated in the mountaintops of Northeast Switzerland were imported in the US during the end of the nineteenth century giving rise to the current Brown Swiss, which is one of the most distributed worldwide breed for dairy production [10]. In addition, the alpine autochthonous breeds are not only essential for their products (many of them specific to a given breed) but also because they are perfectly adapted to the traditional alpine farming system and contribute to the preservation of the rural environment, landscape and ecosystem services [11, 12]. All these features, together with their ancient history make them a relevant part of the alpine culture. In this context, the genetic characterization of such breeds has a prominent role when considering (1) the issues related to strategies for high-quality and sustainable livestock farming that take global warming into account, and (2) that breeds adapted to cold climates are probably more vulnerable.

The availability of genome-wide single nucleotide polymorphism (SNP) data has made it possible to conduct detailed characterizations of the genetic diversity and population structure in cattle. To date, the genetic variability and the relationships between breeds at the genomic level have been investigated on a worldwide scale (e.g. [13, 14]), a regional scale (e.g. [15, 16]), and at the country level (e.g. [17–20]), but a comprehensive study of the Alpine Arc breeds is still lacking. By using genotype data from medium-density SNP arrays, we performed a genome-wide comparative study of 23 cattle populations from the Alpine Arc and three cosmopolitan breeds. We placed particular emphasis on these local breeds to better understand their origin and relationships with cosmopolitan breeds, which have been subjected to worldwide dispersal and intensive human selection for productive purposes. Such an analysis is also important to assess the impact of human selection on these breeds within a known time period and to better design future conservation strategies.

## Methods

### Sampling

**Fig. 1:**
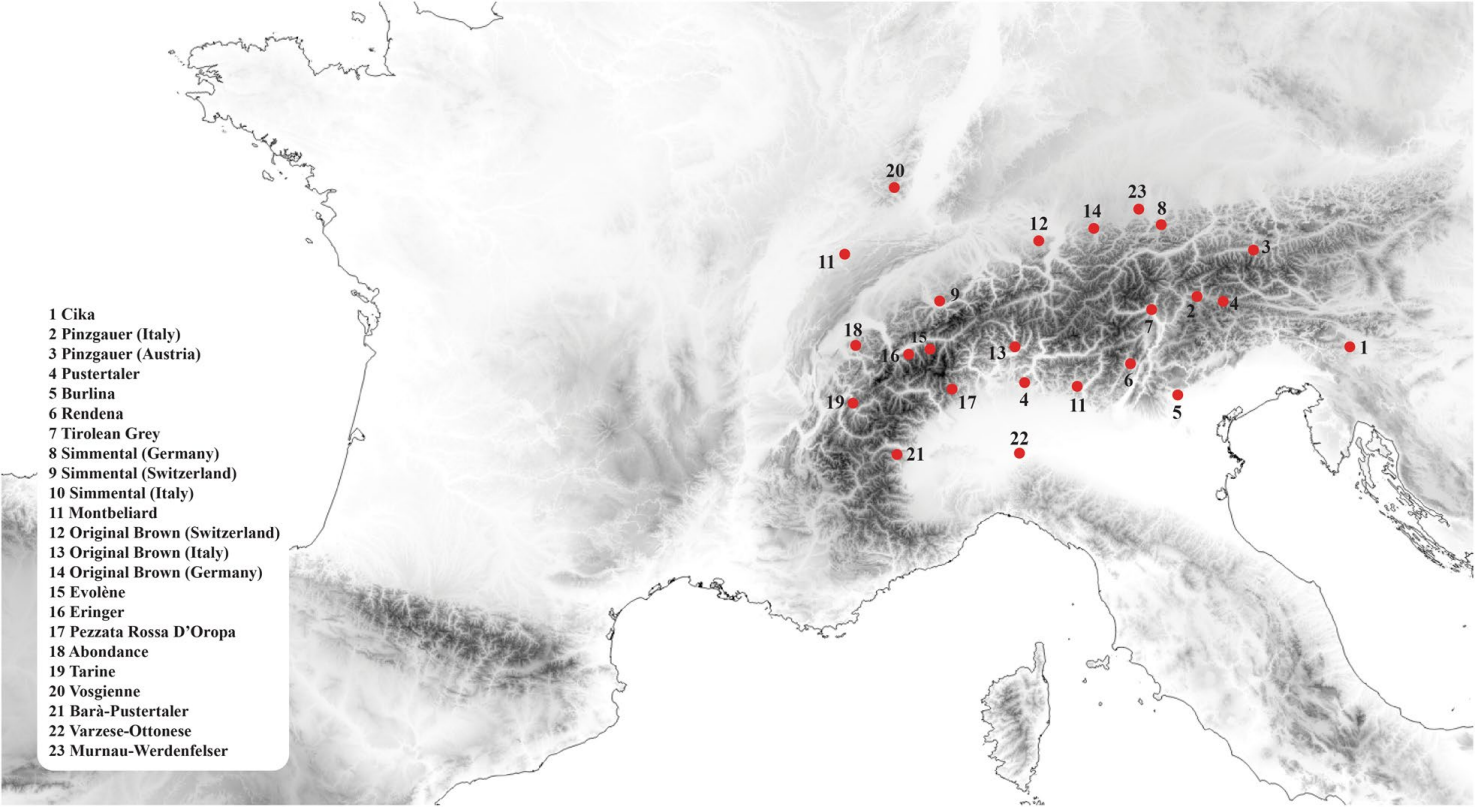
Geographic location of the 23 local cattle breeds from the Alpine Arc analyzed in this study. Cosmopolitan breeds are not shown

A set of 711 individuals belonging to 23 local populations (Fig. 1) and five populations of three cosmopolitan breeds were examined in this study. The local breeds sampled along an eastern/western axis of the Alpine chain included: Cika (CIKA), an autochthonous breed from Slovenia and Croatia; the Italian and Austrian Pinzgauer (PZIT and PZAU, respectively) breed, a dual-purpose breed autochthonous from the Eastern Alps (Austria and Trentino Alto Adige regions); Pustertaler (PUST), an authentic and endangered short-headed breed of cattle that originated from the Puster Valley of the South Tyrol; Barà-Pustertaler (BPUS), a local breed from the Piemonte region whose origin could to be attributed to the Pustertaler breed [21]; Burlina (BURL), a critically endangered breed native from North-East Italy and the Jutland peninsula [22, 23]; Rendena (REND), an indigenous Italian dual-purpose breed that originated from the homonymous valley close to Trent; the Tyrolean Grey (GRTY), a dual purpose breed native of the Tyrol region in Austria; Simmental dual purpose breeds that originated from the Simme Valley and are now widespread throughout the Italian (SIIT), German (SIDE) and Swiss (SISW) side of the Alps (in Italy, Simmental bulls were imported in Friuli since 1870 and, by the 1900s, Simmental completely replaced the local cattle population [24]); Montbéliarde (MONT), a dairy breed from the area of Montbéliard; Original Brown, an endangered breed that originated from Switzerland (OBSW) and is now dispersed in a few areas of the Alpine range including Italy (OBIT) and Germany (OBDS); Evolène (EVOL), an endangered dairy cattle breed from the Valais canton in Switzerland; Eringer (ERIN), a native breed of the Swiss Alps; Pezzata Rossa d’Oropa (PRDO), a breed native from Valle Elvo; Abondance (ABON) that originated in the high valleys of Haute-Savoie; Murnau-Werdenfelser (MAWE), a dairy autochthonous breed from Upper Bavaria; Tarine (TARI), which is restricted to the Susa and Moriana Valleys and descends from the domestic French Tarentaise breed; Varzese-Ottonese (VZOT), a local breed that originated from the Northern side of the Apennine chain; and Vosgienne (VOSG) with a Northern European origin, which was introduced into Switzerland during the seventeenth century. In addition to the indigenous breeds from the Alpine region, we also analyzed three cosmopolitan breeds: two were used as outgroups, i.e. Italian Holstein (HOLS) that originated from the Netherlands and is currently a cosmopolitan dairy breed, and Jersey (JERS), a dairy breed native from Jersey island but currently defined as cosmopolitan; and one breed was included because of its historical relationships with local Alpine breeds, i.e. Brown Swiss, which is represented by three populations because although it derives from a genetic stock of original Brown, it was imported to the USA, strongly selected for milk production and subsequently reintroduced to worldwide countries including Italy (BRIT), Germany (BRDE) and Switzerland (BRSW).

Sample size and locations of all the cattle breeds/populations included in this study are in Table 1 and the origins of the samples of the indigenous breeds from the Alpine Arc are shown on Fig. 1.

**Table 1:** Genetic diversity indices for the analyzed cattle breeds

Breed	Number	Code	H_o_ ± SD	H_e_ ± SD	MAF ± SD	N_e_
Italian Holstein	32	HOLS	0.342 ± 0.171	0.336 ± 0.154	0.305 ± 0.209	77
Cika	26	CIKA	0.356 ± 0.163	0.347 ± 0.141	0.277 ± 0.163	107
Pinzgauer (Italy)	24	PZIT	0.350 ± 0.172	0.337 ± 0.149	0.282 ± 0.179	76
Pinzgauer (Austria)	30	PZAU	0.351 ± 0.163	0.342 ± 0.144	0.280 ± 0.173	122
Pustertaler	24	PUST	0.342 ± 0.185	0.324 ± 0.16	0.276 ± 0.180	62
Burlina	24	BURL	0.353 ± 0.167	0.345 ± 0.145	0.292 ± 0.185	83
Rendena	24	REND	0.334 ± 0.176	0.327 ± 0.156	0.274 ± 0.188	82
Tyrolean Grey	30	GRTY	0.335 ± 0.169	0.332 ± 0.152	0.272 ± 0.179	93
Simmental (Germany)	30	SIDE	0.339 ± 0.168	0.332 ± 0.151	0.269 ± 0.175	127
Simmental (Switzerland)	20	SISW	0.342 ± 0.177	0.330 ± 0.152	0.270 ± 0.178	92
Simmental (Italy)	31	SIIT	0.342 ± 0.167	0.333 ± 0.149	0.270 ± 0.174	113
Montbéliard	20	MONT	0.337 ± 0.193	0.316 ± 0.163	0.270 ± 0.198	60
Brown Swiss (Germany)	30	BRDE	0.319 ± 0.179	0.311 ± 0.163	0.270 ± 0.190	79
Brown Swiss	19	BRSW	0.320 ± 0.191	0.307 ± 0.167	0.268 ± 0.206	56
Brown Swiss (Italy)	32	BRIT	0.309 ± 0.185	0.301 ± 0.168	0.266 ± 0.211	65
Original Brown (Switzerland)	20	OBSW	0.338 ± 0.176	0.331 ± 0.151	0.272 ± 0.179	81
Original Brown (Italy)	18	OBIT	0.348 ± 0.179	0.334 ± 0.149	0.278 ± 0.183	90
Original Brown (Germany/Switzerland)	35	OBDS	0.338 ± 0.166	0.332 ± 0.151	0.270 ± 0.177	124
Evolène	21	EVOL	0.311 ± 0.185	0.309 ± 0.166	0.271 ± 0.206	59
Eringer	36	ERIN	0.333 ± 0.169	0.327 ± 0.155	0.270 ± 0.183	130
Pezzata Rossa D’Oropa	23	PRDO	0.335 ± 0.176	0.329 ± 0.154	0.271 ± 0.181	80
Abondance	20	ABON	0.346 ± 0.189	0.323 ± 0.159	0.272 ± 0.190	68
Tarine	18	TARI	0.336 ± 0.185	0.325 ± 0.158	0.272 ± 0.188	72
Vosgienne	20	VOSG	0.347 ± 0.178	0.335 ± 0.15	0.271 ± 0.174	75
Barà-Pustertaler	24	BPUS	0.351 ± 0.164	0.344 ± 0.142	0.275 ± 0.165	94
Varzese-Ottonese	30	VZOT	0.348 ± 0.164	0.341 ± 0.146	0.279 ± 0.174	87
Murnau-Werdenfelser	30	MAWE	0.349 ± 0.179	0.328 ± 0.156	0.274 ± 0.186	73
Jersey	20	JERS	0.297 ± 0.198	0.286 ± 0.176	0.286 ± 0.246	56

Observed (H_o_) and expected (H_e_) heterozygosity, average minor allele frequency (MAF), effective population size (N_e_) and standard deviation (SD)

### Genotyping and quality control

For all the aforementioned breeds, SNP data that were obtained with the Bovine SNP50K BeadChip, were collected from previous studies, except for Original Brown from Italy (OBIT), as reported in Additional file 1: Table S1. These data were merged with PLINK [25] and the autosomal SNPs that were common to all datasets were retained. After merging, the SNP data were pruned based on minor allele frequency (MAF) and on missing call rate both per SNP and per individual (− maf 0.05, − geno 0.01 and − mind 0.1) using PLINK [25]. Finally, 30,176 SNPs remained in the genotyping dataset for further analyses.

### Genetic diversity and runs of homozygosity

PLINK [25] was also used to estimate observed (H_o_) and expected (H_e_) heterozygosities and MAF. Trends in historical effective population size (N_e_) based on linkage disequilibrium (LD) data were estimated by using the program SNeP v1.1 [26].

Runs of homozygosity (ROH) were detected as described in Mastrangelo et al. [19], using a sliding window approach of 50 SNPs in PLINK. For each animal, inbreeding based on ROH (*F*_ROH_) was calculated as the proportion of the genome in ROH over the overall length of the genome covered by SNPs (2,541,174 kb).

### Population structure and admixture

Pairwise genetic relationships were estimated using a matrix of genome-wide identity-by-state (IBS) genetic distances calculated by PLINK [25] and plotted using a multidimensional scaling (MDS) plot.

To examine population structure, we used the maximum likelihood clustering approach as implemented in the software ADMIXTURE [27], which estimates the individual ancestry proportions given a K number of ancestral populations. We performed three independent runs with K ranging from 2 to 28, using the default parameters and a cross-validation to 10-fold (− cv = 10). To check for convergence, we compared parameters across runs by assessing when the increase in log-likelihood between iterations was less than 10^−4^. Then, the obtained **Q** matrix with membership coefficients was used to visualize results in a circular fashion using the *membercoef.circos* function in the R package BITE [28].

We used the ARLERQUIN software [29] to estimate population relatedness using pairwise estimates of *F*_ST_ between all analyzed breeds. Phylogenetic relationships between populations were explored based on Reynolds genetic distances. A neighbor-net graph was constructed from the estimated genetic distances using the SPLITSTREE software [30].

To infer the presence of patterns of population splitting and introgression events within a phylogenetic framework, we used the software TREEMIX [31], which implements a maximum likelihood approach based on allele frequencies. The genotyping data of the Baoule cattle from Burkina Faso [14] was used as outgroup. First, we generated a maximum likelihood tree to reconstruct the relationships between all the breeds included in this study without migration events. Subsequently, we performed five iterations by taking linkage disequilibrium across blocks of 500 contiguous SNPs into account and incorporating admixture events (E) ranging from 0 to 10. The best predictor for number of migration edges was selected using the *optM* function in the R package OptM [32].

## Results

### Genetic diversity and genomic inbreeding

Genetic diversity indices for the 28 populations analyzed are in Table 1. H_o_ ranged from 0.297 ± 0.198 to 0.356 ± 0.163 and H_e_ ranged from 0.286 ± 0.176 to 0.347 ± 0.141; both minimum and maximum values for H_o_ and H_e_ were obtained for the Jersey and Cika breeds, respectively. The lowest and highest mean values of MAF were obtained for Italian Brown (0.266 ± 0.211) and Italian Holstein (0.305 ± 0.209), respectively. The effective population size (N_e_) estimates relating to the 13th generation (assumed to represent the contemporary N_e_) for each breed are in Table 1. The minimum N_e_ value (56) was found for Brown Swiss and Jersey, and the maximum value (130) for Eringer. For the three populations of the selected Brown Swiss breed (BRIT, BRSW and BRDE), N_e_ was small (on average 60.5) compared to that of the original Brown breeds (OBIT, OBDS and OBSW) (on average 98.3).

**Fig. 2:**
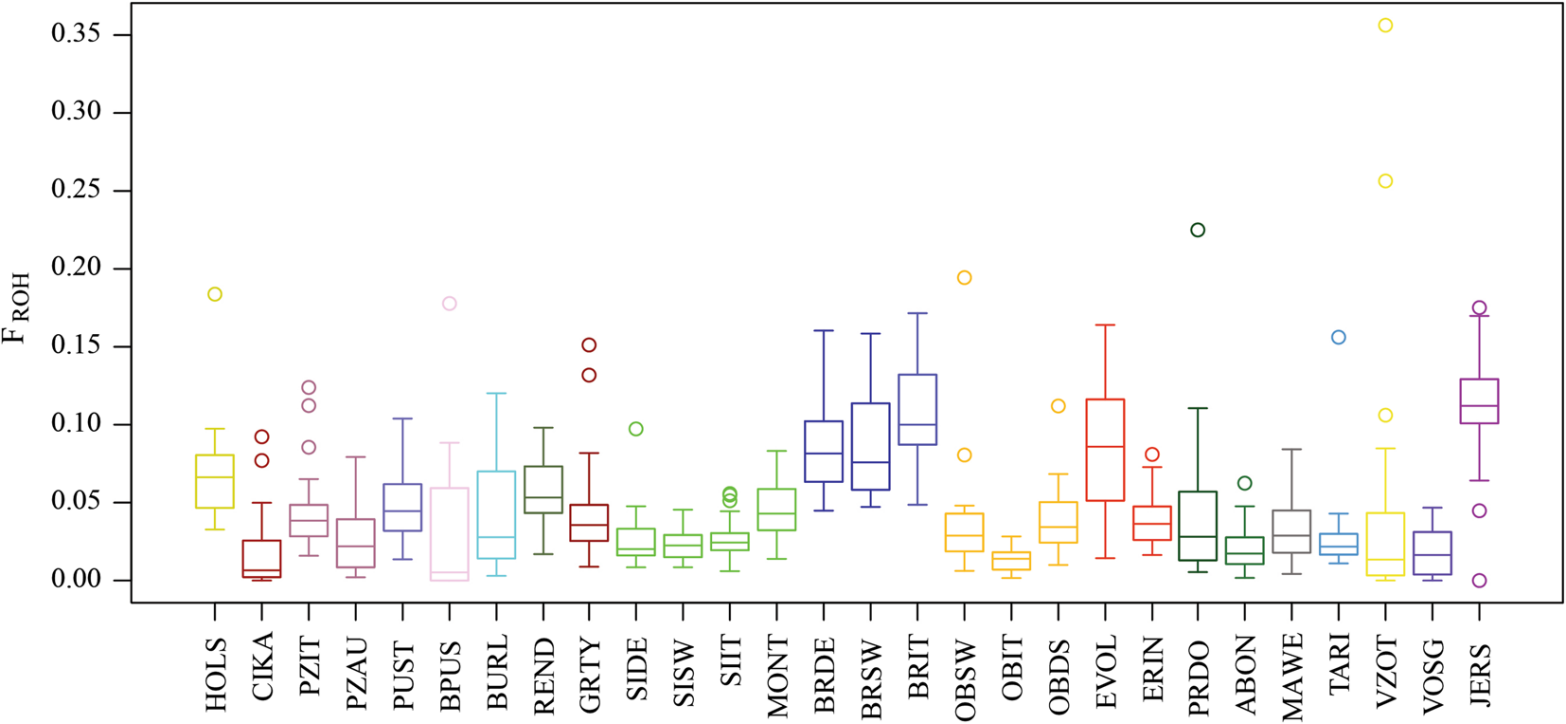
Inbreeding coefficients (*F*_ROH_) inferred from runs of homozygosity for each breed. For a full definition of breeds, see Table 1

Individual genomic inbreeding was evaluated using ROH data. ROH inbreeding coefficients are reported in Fig. 2. The highest mean value of *F*_ROH_ was observed in Jersey (0.110), modern Brown (0.105) and Evolène (0.088) breeds, whereas the lowest values were found for Original Brown from Italy (OBIT) (0.014) and Cika (0.017) and, to a lesser extent, for the Simmental group (SIIT, SIDE, SIMM) (~ 0.023) with the notable exception of the Montbéliarde breed (0.045). In general, *F*_ROH_ were higher for the highly selected cosmopolitan breeds such as Jersey and Holstein compared to most of the autochthonous breeds.

### Population structure and admixture

**Fig. 3:**
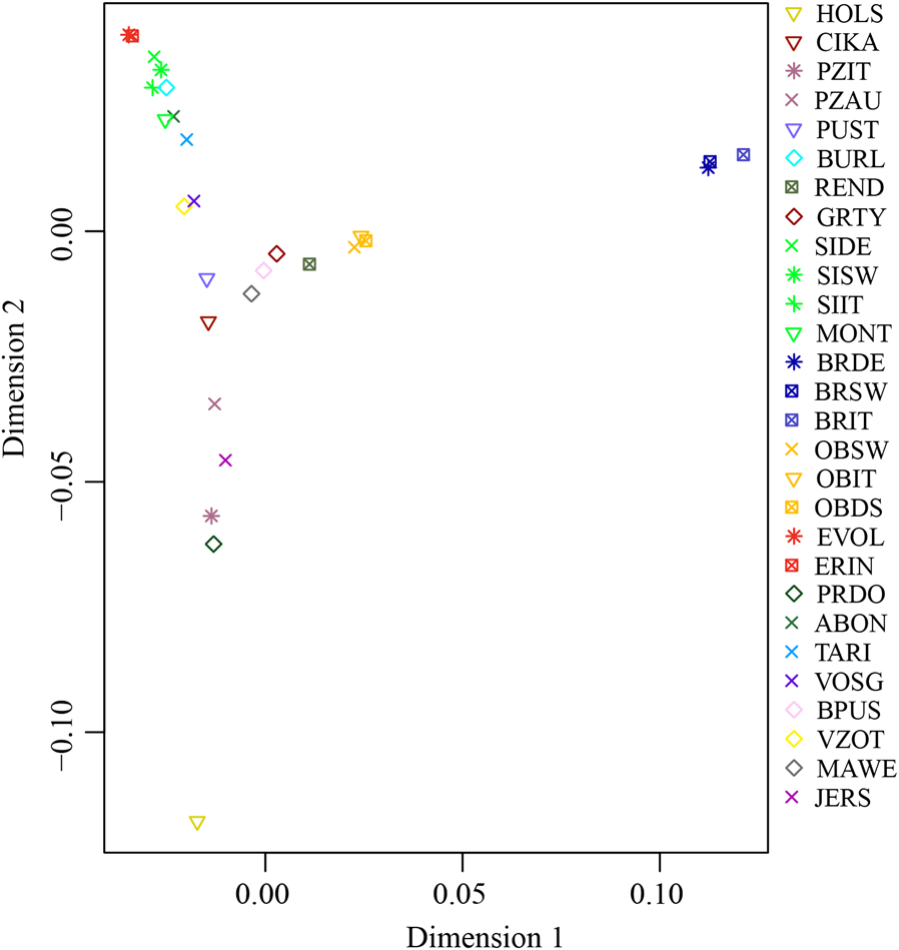
Genetic relatedness of cattle breeds using a multidimensional scaling (MDS) approach. The first two dimensions, C1 and C2, explained 3.2 and 2.3%, respectively, of the total variation. For a full definition of breeds, see Table 1

In the multidimensional scaling (MDS) analysis, the first two dimensions explained 3.2 and 2.3% of the total variance, respectively. The first dimension (D1) showed a marked separation between the selected Brown breeds (BRIT, BRSW, and BRDE) and all the other breeds, whereas the second dimension (D2) clearly differentiated the Holstein breed (Fig. 3). In this dimension, a geographic gradient along the Alpine East–West axis was found. Most of the breeds from the Western and Central Alps are distributed mainly at the bottom right side of the MDS plot, whereas the breeds from Eastern Alps are distributed in the central portion of the MDS plot (Fig. 3). In addition, the MDS analysis confirmed the genetic identity of several populations belonging to the same breed but raised in different areas such as the three Original Brown from Italy (OBIT), Germany (OBDE) and Switzerland (OBSW), as well as the Eringer and Evolène breeds, which displayed almost overlapping centroids. However, although for the two Pinzgauer populations from Italy and Austria (PZIT and PZAU) no overlapping centroids were observed, they were very close to each other. With the other dimensions in the MDS analysis, its discrimination power decreased progressively for the main breeds analyzed here (data not shown).

**Fig. 4:**
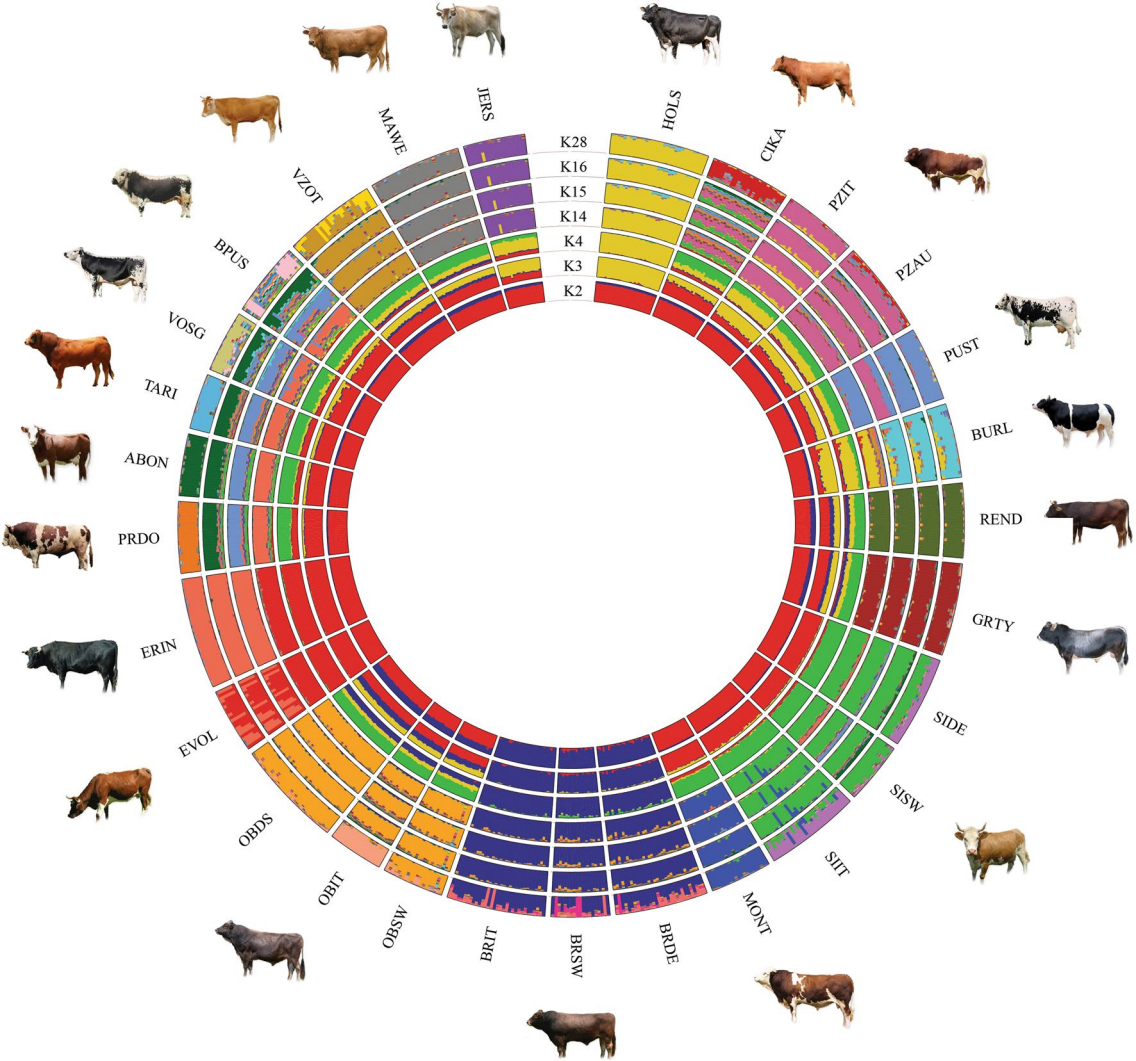
Admixture analysis plot in a circular fashion. The most significant values of K (number of genetic clusters) according to the cross-validation parameters are shown. For a full definition of breeds, see Table 1

For the population structure inferred by using ADMIXTURE with K ranging from 2 to 28, cross-validation values showed almost identical CV errors from K = 16 onwards (see Additional file 2: Figure S1). The ADMIXTURE plot with all K values from 2 to 28 is reported in Additional file 3: Figure S2. The ADMIXTURE components at K = 2 highlighted a separation between the modern Brown breeds (BRIT, BRDE, BRSW) and all the other breeds (Fig. 4). At K = 3, the Holstein breed forms a separate group, and at K = 4 a component shared by all the Simmental breeds (SIDE, SIIT, SISW, MONT) becomes evident. Except for the modern Brown breeds, Eringer, and Evolène, such a Simmental component is present with various proportions in all the other breeds with the smallest proportion observed for the Jersey and Burlina breeds. At K = 16, almost all the groups of breeds have their own identity but, at this value, some breeds show a moderate level of admixture (BPUS, CIKA, PRDO, TARI, VOSG). Finally, at K = 28 which corresponds to the number of populations analyzed in this study, admixture events emerge especially in the selected Brown (BRSW, BRDE, BRIT) and in the Simmental group (SIDE, SISW, SIIT).

Genetic differentiation between all pairs of populations was estimated by the *F*_ST_ statistics (see Additional file 4: Table S2), and the values confirm the results of the MDS and ADMIXTURE analyses, i.e. the highest *F*_ST_ values were obtained for pairwise comparisons that included the breeds used as outgroup (HOLS and JERS) and the modern Brown breeds. For example, an *F*_ST_ of 0.193 was found between Jersey and Italian Brown, whereas it was only 0.001 between the modern Brown breeds.

**Fig. 5:**
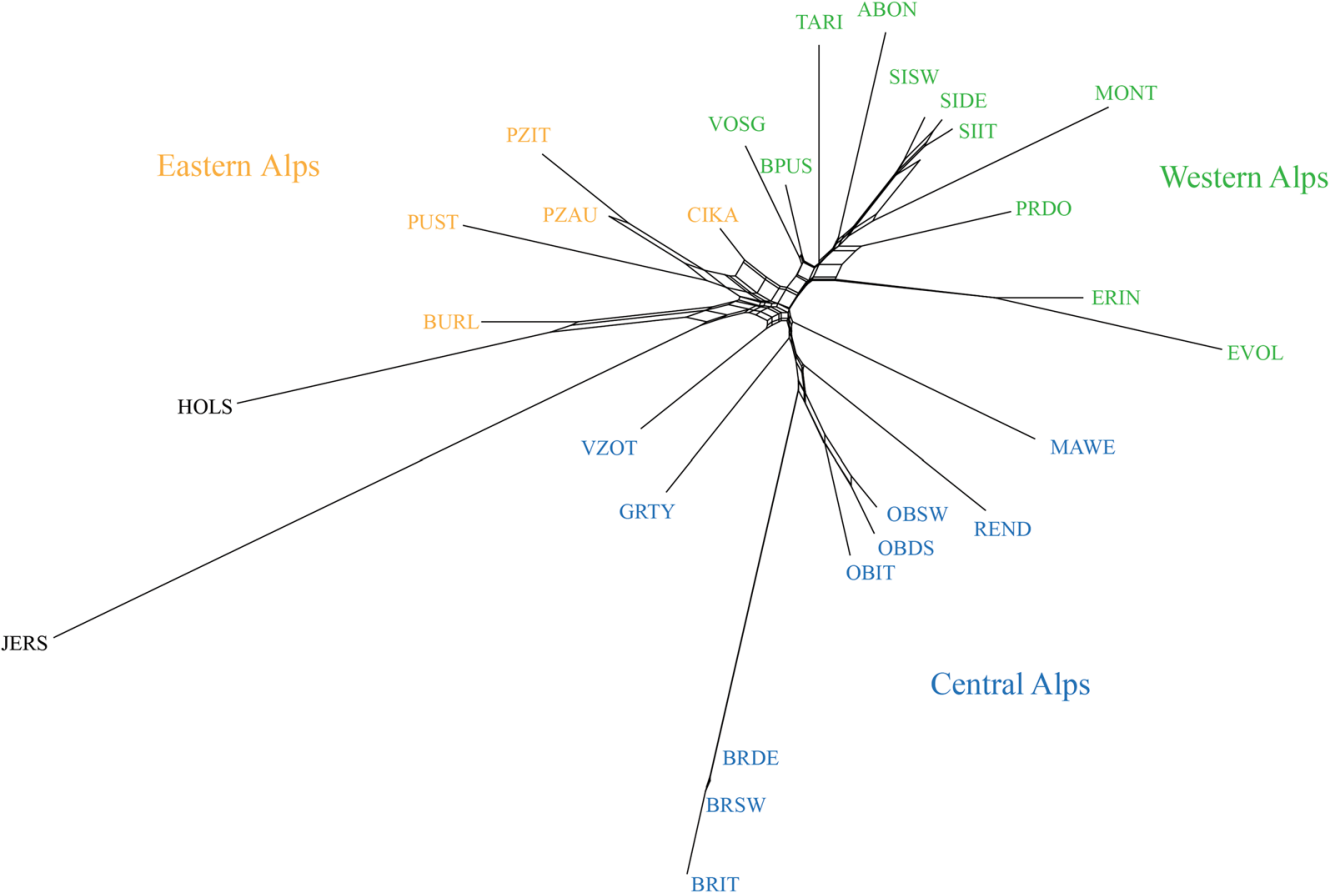
Neighbor-net graph based on Reynolds genetic distances. The color of breed codes highlights the main geographic clusters: green for Western Alps, orange for Eastern Alps and blue for Central Alps. For a full definition of breeds, see Table 1

The neighbor-net graph, which was constructed based on Reynold’s genetic distances between pairs of breeds, showed several geographically-consistent groups of local breeds (Fig. 5): (i) a group including breeds from the Eastern Alps (CIKA, PZAU, PZIT and PUST); (ii) a second group including breeds mostly from the Central Alps (BRIT, BRSW, BRDE OBIT, OBDS, OBSW, REND, MAWE, GRTY and VZOT) and within this group a clear differentiation in terms of branch length is detected between the Original and the modern Brown breeds (Fig. 5); (iii) a third group with a Western Alps gravitation including all Simmental-derived breeds (SIDE, SIIT, SISW and MONT), the Barà-Pustertaler, the French (ABON, VOSG and TARI), the Pezzata Rossa d’Oropa and the Swiss breeds (ERIN and EVOL).

**Fig. 6:**
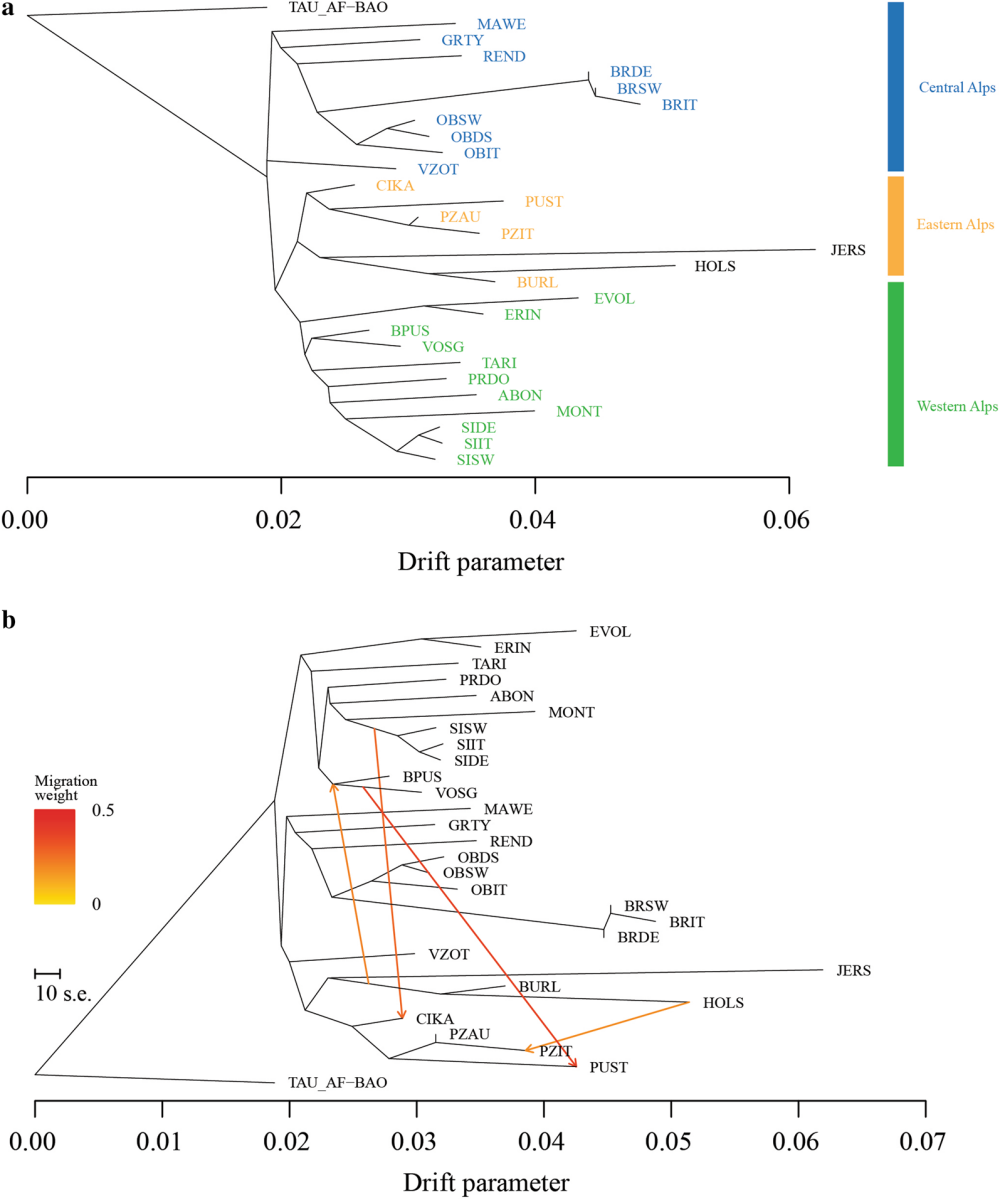
Maximum likelihood phylogenetic tree inferred using TreeMix with no migration event (**a**) and with four migration edges allowed (**b**). The Baoule cattle (TAU_AF-BAO) was used as an outgroup. For a full definition of breeds, see Table 1

Consistent with both the MDS plot and the neighbor-net graph, the TREEMIX phylogram without migration events shows a clear distribution of clusters according to the geographic origin of breeds (Fig. 6a). This topology remained fairly stable across iterations and also when migration events were taken into account (Fig. 6b). The SiZer method implemented in the optM function (R package OptM) indicated a major changing point in the log likelihood at four migration events (see Additional file 5: Figure S3), i.e. a stronger signal of admixture between the Vosgienne and the Pustertaler breeds and between the Simmental group and the Cika breed. Two additional migration events, although with a weaker signal of admixture, were inferred, one between Holstein and Pinzgauer from Italy (PZIT), and the other connecting the Burlina and Holstein breeds with the Vosgienne and Barà-Pustertaler breeds (Fig. 6b).

## Discussion

### Relationships between the cosmopolitan and autochthonous breeds

The results based on genetic diversity indices, multidimensional scaling, ROH and ADMIXTURE analyses showed convergence, which indicates that most of the breeds have their own genetic signature. Furthermore, we detected a genetic structure according to the geographic origin of the local breeds with cases of both latitudinal and longitudinal admixture events. In spite of this geographic consistency, the modern Brown (BRSW, BRIT and BRDE) populations were part of the most differentiated breeds, and both the multidimensional scaling (MDS) and ADMIXTURE analyses showed that they were clearly separated from all the other breeds. Such a strong genetic divergence of the modern Brown Swiss from other Alpine breeds including the original Brown was previously reported [15, 33, 34], which is quite interesting considering both their Alpine origin and their relatively recent separation. However, genetic diversity indices and effective population sizes (N_e_) were low as expected when strong genetic drift occurs (Table 1). In addition, based on the ROH analysis, the modern Brown Swiss breeds had higher *F*_ROH_ (Fig. 2). One possible explanation for such a pattern is the combination of a founder effect promoted by the narrow genetic base from which they originated with different selective trajectories to which the original and the modern Brown Swiss breeds have been subjected. Indeed, the modern Brown Swiss breeds are known to have originated from a few individuals (about 155) [10] that were imported into the US at the end of the nineteenth century and at the beginning of the twentieth century. Moreover, the homogeneity observed within the modern Brown Swiss breeds suggests that there have been efforts to maintain these breeds as pure as possible.

In general, we found that, compared to some local breeds, the cosmopolitan breeds had lower genetic diversity indices and N_e_. Usually, high selection pressure and the use of artificial insemination are the main reasons for a low level of genetic diversity. In fact, apart from the modern Brown Swiss breeds, the Jersey and, to a lesser extent, Holstein breeds had the smallest N_e_. The results of the ROH analysis also confirmed the above findings, i.e. *F*_ROH_ values were high for the highly selected cosmopolitan breeds (Holstein, Jersey and Brown Swiss) (Fig. 2).

The second dimension in the MDS analysis also discriminates Holstein and to a lesser extent, Jersey, from all the other breeds. Although previously reported [19], the divergent pattern between the Holstein and modern Brown Swiss breeds is unexpected considering that both of them have been subjected to selection for milk production, which suggests that these genes have little impact on the overall genomic structure. The result of the MDS analysis parallels the genetic pattern inferred with ADMIXTURE at K = 3, which separates the Holstein breed from all the other breeds. However, N_e_ was larger for the Simmental derived dual-purpose breeds (SIDE, SISW and SIIT) except for the Montbéliarde breed (N_e_ = 60). This is also confirmed by the ROH analysis that showed that the Montbeliarde had higher values than the Simmental derived breeds. The Montbéliarde breed originates from the Swiss Simmental breed from which it diverged approximately 150 years ago [35]. However, a study based on the polymorphism of immunogenetic markers highlighted a close relationship between Montbéliarde and some eastern French breeds such as the Vosgienne, Tarine and Abondance [36]. It should be noted that some individuals of the Italian Simmental showed traces of Montbéliarde introgression starting from K = 10 (Fig. 4, Additional file 3: Figure S2). The history of the Simmental breed dates back to the Middle Ages and is believed to result from a cross between large German cattle and a small Swiss indigenous breed [37], which might also explain the Simmental ancestral component at K = 4 (in green), that we found for most of the Alpine breeds (Fig. 4).

### Genetic overview of the autochthonous Alpine breeds

Genetic diversity is an intrinsic factor that influences the adaptive capacity and resilience of populations. Across all the breeds analyzed here, the local breeds showed high or intermediate values of genetic diversity indices and N_e_. In all cases, N_e_ was above 50, which is the threshold for a breed/population to be considered at risk [38]. Among the local breeds from the Eastern Alps, all the breeds except Pustertaler had large N_e_ and those with the largest N_e_ were Cika and Austrian Pinzgauer (107 and 122, respectively); these values being larger than those calculated for the same breeds in a previous work but with a reduced SNP array [39]. Natural service is still frequent in these local populations, which requires the use of more sires at each generation. Therefore, although these are closed populations, this practice increases the genetic diversity within the population and thus decreases genomic inbreeding [18]. The genetic relatedness of the breeds from the Eastern Alps, as highlighted by the shared ancestral components (Fig. 4) is probably related to their geographic proximity. Moreover, introgression of Pinzgauer into Cika cattle is ongoing since the first half of the twentieth century to improve its coat color with white stripes [40–42]. Concerning the Pustertaler breed, the small N_e_, i.e. 62 found here, is in line with the total population size estimated at 60 individuals in 1994, which explains the status of this breed as critically-maintained [43]. Because excessive inbreeding can reduce the long-term fitness of a population, it is necessary to preserve this population and prevent further increase of inbreeding. The ADMIXTURE analysis showed an important ancestral Simmental component but also shared ancestry with both the Pinzgauer and some Italian (PRDO and BPUS) and French western breeds (ABON, TARI and VOSG). Cross-breeding between the Pustertaler and both Simmental and Pinzgauer cattle have been reported by Felius [44], while the origin of this breed from the French Vosges breeds might explain its relatedness with western breeds [45, 46]. The autochthonous Burlina breed from Eastern sub-Alpine regions is classified as critically endangered (FAO Web site: http://dad.Fao.org/en/Home.htm) because of its reduced population size, but we found moderate estimates of N_e_ (83) and *F*_ROH_ (0.041). Although Burlina has been reported to have a common origin with the Black Pied cattle group, we did not find any trace of such an introgression. Instead, our admixture analysis supports previous results based on microsatellite data indicating a close relationship with the Holstein breed [22, 34]. In fact, the Burlina breed originated in the North Sea area from which Holstein also derived and crossbreeding between these two breeds are historically reported [47]. N_e_ and *F*_ROH_ values for the Tyrolean Gray breed were also intermediate, 93 and 0.043, respectively. However, whereas Burlina showed an admixed component especially from Holstein, the Tyrolean Grey breed displayed a more homogeneous pattern, which could be due to many within-population crosses because the number of individuals greatly decreased after World War I [48]. Local breeds from the Western Alps also showed moderate to large N_e_ values and generally low *F*_ROH_ values, with the largest and smallest N_e_ found for Eringer (130) and Evolène (59), respectively. Our result confirms that of Flury et al. [49] for the Eringer breed and provides additional support that these populations have benefited from proper breed management. The low genetic diversity of the Evolène breed is in agreement with the previous studies of Del Bo et al. [34] using microsatellite loci and Signer-Hasler et al. [18] using an SNP array and is explained by its critical-maintained status, since it was recovered after its size-contraction subsequent to World War II [38]. In fact, this population is specific to a very small region and its census is less than 200 registered herd-book cows [18]. Therefore, our findings raise the possibility of a risk for the genetic diversity of some local cattle breeds, such as Evolène, and their decrease in N_e_ needs to be monitored and taken into account. This low genome-wide genetic variability could also be related to a lower adaptation potential, which could represent a threat to the long-term persistence of such breeds [19]. In general, the genetic diversity indices, the effective population size and the genomic inbreeding levels were congruent with the protection status of the Alpine local cattle breeds based on their reduced demographic size.

Within the autochthonous breeds from Western Alps, we found two major genetic groups, one including the Swiss (EVOL and ERIN) and the other including the French (ABON, MONT, TARI), the Barà-Pustertaler, the Vosgienne, the Pezzata Rossa d’Oropa and the derived-Simmental breeds (SISW, SIIT and SIDE) (Figs. 5, 6). According to Bettini [50], in the Western Alps during the second half of the nineteenth century, there was a variety of breeds of foreign origin such as the Tarine (the Tarantaise of Savoy and Isère French valleys), the Simmental and Hèrens breeds (from Switzerland) and a wide range of their crosses. This might explain the closer relationship between the Pezzata Rossa D’Oropa and the French and Simmental breeds. Furthermore, this is very much in line with the documented origin of the Pezzata Rossa D’Oropa, which results from a cross between Simmental and Valdostana cattle, because they share a similar morphology and red pied color [38]. In addition, our results shed new light on the debated origin of the Barà-Pustertaler breed. Currently, this breed is reared in the Piemonte region (Western Alps) and there are two main hypotheses that explain its origin, one arguing a Wasler German origin which might relate this breed to the German Pustertaler and the other to the French Vosgienne with which it shares phenotypic traits. Our results indicate a closer genetic relationship with the French breeds (VOSG) than with the Pustertaler as highlighted by Battaglini et al. [21].

Our phylogenetic reconstruction, which includes all the analyzed populations and no migration event, conforms largely to the ADMIXTURE analysis. However, the tree topologies as inferred in TREEMIX allowed us to better clarify the relationships among some of the breeds. For example, the Rendena cattle, which is an old native breed from the homonymous valley in the Eastern Alps, is closer to the Brown breeds than to the eastern breeds, as also highlighted by Del Bo et al. [34]. This could be related to different political and cultural administrations between the Rendena Valley and the Sud Tirol, which became annexed to Italy only in 1919. Moreover, contrary to the ADMIXTURE analysis which indicated an early separation of the modern Brown Swiss breed at K = 2, the phylogenetic tree placed the modern Brown Swiss as sister of the Original Brown. Finally, migration events indicated concordant signals, which confirms most of the admixture events already reported in the literature, and highlighted in our ADMIXTURE analysis. For example, the inferred gene flow between the Simmental and Cika breeds has been documented since 1962 in West Slovenia [40], and the gene flow between the Vosgienne and the Pustertaler breeds might be related to the eastern origin of the Barà-Pustertaler breed and its subsequent cross with the French Vosgienne breed. Finally, Kidd and Pirchner [51] highlighted for the first time the gene exchange between the Holstein and Italian Pinzgauer (PZIT) breeds, which was later confirmed by Mastrangelo et al. [19]. Based on our results, it is difficult to conclude whether the observed Holstein introgression into the Pinzgauer breed reflects a recent crossbreeding or alternatively indicates a shared ancestral component. However, it should be noted that the Pinzgauer breed derives from cattle that were brought by Germanic settlers from the north during the Middle Ages, which indicates that a common area of origin for these two breeds is plausible.

## Conclusions

Our study represents one of the most comprehensive genetic diversity analysis of the Alpine cattle breeds, to date. Such a dataset, including most of the local breeds present in this region, has allowed us to expand knowledge on the evaluation and status of Alpine cattle diversity. Indeed, an important prerogative in conservation genetics and genetic improvement of breeds should be the maintenance of local animal genetic diversity. Local breeds may be more prone to extinction, which leads to a reduced overall adaptive genetic potential, and this is crucial for the recovery of particular selection strategies. Therefore, monitoring the genetic diversity of these local breeds for conservation purposes is fundamental to meet future breeding needs, especially in the context of global climate change. Interestingly, we found a remarkable genetic relationship among the breeds that originate from the same geographical area. However, in spite of past admixture events, several of these local breeds have conserved distinctive characteristics; they can be clearly discriminated, which is probably due to differences in genetic origin and geographic location. Our analyses showed that the autochthonous breeds from the Alpine Arc encompass an important component of the European cattle genetic diversity. This is especially interesting since local breeds represent an important cultural component that contributes to the maintenance of the alpine rural environment. In general, local or marginal populations are expected to be more prone to genetic erosion or inbreeding phenomena, which may be deleterious. Indeed, all the analyzed breeds in this study showed high or intermediate values of genetic diversity, which are essential for implementing breeding actions and conservation strategies. In this context, genomic information should have a crucial role in assisting the management of small populations.

## Supplementary information

**Additional file 1: Table S1.** Name of the breeds, sample size, breed codes and source of genotyping data.

**Additional file 2: Figure S1.** Cross-validation plot of admixture analysis for all values of K (number of clusters) ranging from 2 to 28.

**Additional file 3: Figure S2.** Admixture analysis plot in a circular fashion with all values of K (number of clusters) ranging from 2 to 28.

**Additional file 4: Table S2.** Fixation indices (*F*_ST_) between all pairs of breed populations analyzed in this study.

**Additional file 5: Figure S3.** Increment in the log likelihood for all tested migration events calculated by using the optM function in the R package OptM.

## Data Availability

The data that support the findings of this study are available on request from the corresponding author, except for the genotyping data of Evolène and Eringer breeds.
