# Supplementary material for: The genetic heritage of Alpine local cattle breeds using genomic SNP data
Source: Genet Sel Evol. 2020 Jul 14;52:40. doi: 10.1186/s12711-020-00559-1 (PMC7362560; doi:10.1186/s12711-020-00559-1)
Supplement: Supplementary file 1 — Additional file 1: Table S1. Name of the breeds, sample size, breed codes and source of genotyping data. [file 12711_2020_559_MOESM1_ESM.doc]

**Table S1.** Name of the breeds, sample size (N), breed codes and source of genotyping data.

| **Breed** | **N** | **Code** | **Geographic Origin** | **Data origin** |
| --- | --- | --- | --- | --- |
| Italian Holstein | 32 | HOLS | Italy | Mastrangelo *et al.* [19] |
| Cika | 26 | CIKA | Slovenia | Ramljak *et al.* [6] |
| Pinzgauer (Italy) | 24 | PZIT | Italy | Mastrangelo *et al.* [19] |
| Pinzgauer (Austria) | 30 | PZAU | Austria | Decker *et al.* [14]; Ramljak *et al.* [6] |
| Pustertaler | 24 | PUST | Italy | Mastrangelo *et al.* [19] |
| Burlina | 24 | BURL | Italy | Mastrangelo *et al.* [19] |
| Rendena | 24 | REND | Italy | Mastrangelo *et al.* [19] |
| Tyrolean Grey | 30 | GRTY | Austria | Ramljak *et al.* [6] |
| Simmental (Germany) | 30 | SIDE | Germany | Ramljak *et al.* [6] |
| Simmental (Switzerland) | 20 | SISW | Switzerland | Decker *et al.* [14] |
| Simmental (Italy) | 31 | SIIT | Italy | Mastrangelo *et al.* [19] |
| Montbeliard | 20 | MONT | France | Gautier *et al.* [13] |
| Brown Swiss (Germany) | 30 | BRDE | Germany | Ramljak *et al.* [6] |
| Brown Swiss | 19 | BRSW | Americas | Gautier *et al.* [13] |
| Brown Swiss (Italy) | 32 | BRIT | Italy | Mastrangelo *et al.* [19] |
| Original Brown (Switzerland) | 20 | OBSW | Switzerland | Decker *et al.* [14] |
| Original Brown (Italy) | 18 | OBIT | Italy | This study |
| Original Brown (Germany/Switzerland) | 35 | OBDS | Switzerland/Germany | Ramljak *et al.* [6] |
| Evolène | 21 | EVOL | Switzerland | Signer-Hasler *et al.* [18] |
| Eringer | 36 | ERIN | Switzerland | Signer-Hasler *et al.* [18] |
| Pezzata Rossa D’Oropa | 23 | PRDO | Italy | Mastrangelo *et al.* [19] |
| Abondance | 20 | ABON | Southeast France | Gautier *et al.* [13] |
| Tarine | 18 | TARI | Southeast France | Decker *et al.* [14] |
| Vosgienne | 20 | VOSG | Northeast France | Decker *et al.* [14] |
| Barà-Pustertaler | 24 | BPUS | Italy | Mastrangelo *et al.* [19] |
| Varzese-Ottonese | 30 | VZOT | Italy | Mastrangelo *et al.* [19] |
| Murnau-Werdenfelser | 30 | MAWE | Germany | Ramljak *et al.* [6] |
| Jersey | 20 | JERS | Jersey Island | Decker *et al.* [14] |
