# Supplementary figures and images for: The genetic heritage of Alpine local cattle breeds using genomic SNP data
Source: Genet Sel Evol. 2020 Jul 14;52:40. doi: 10.1186/s12711-020-00559-1 (PMC7362560; doi:10.1186/s12711-020-00559-1)

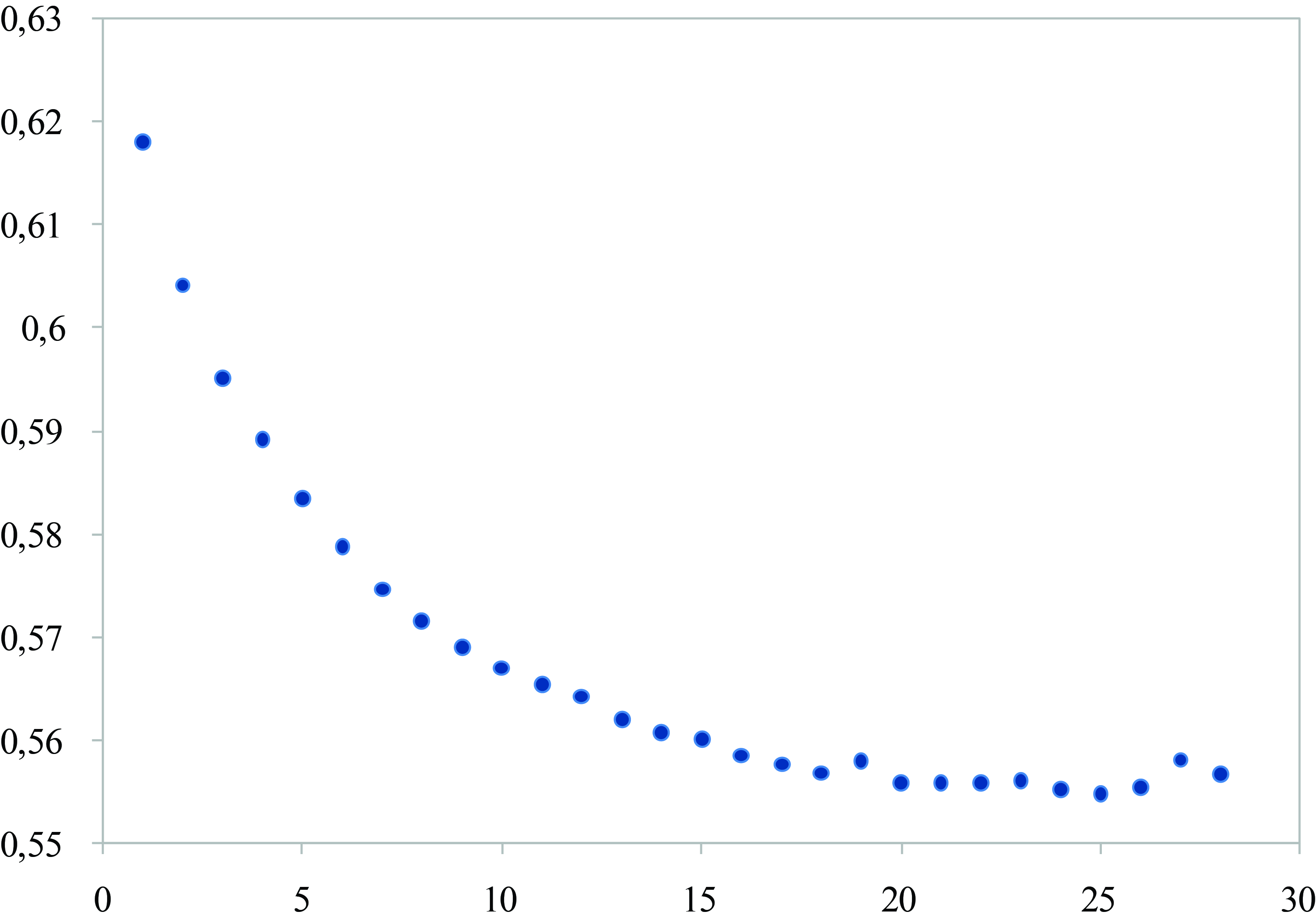

Supplement: Supplementary file 2 — Additional file 2: Figure S1. Cross-validation plot of admixture analysis for all values of K (number of clusters) ranging from 2 to 28. [file 12711_2020_559_MOESM2_ESM.jpg]

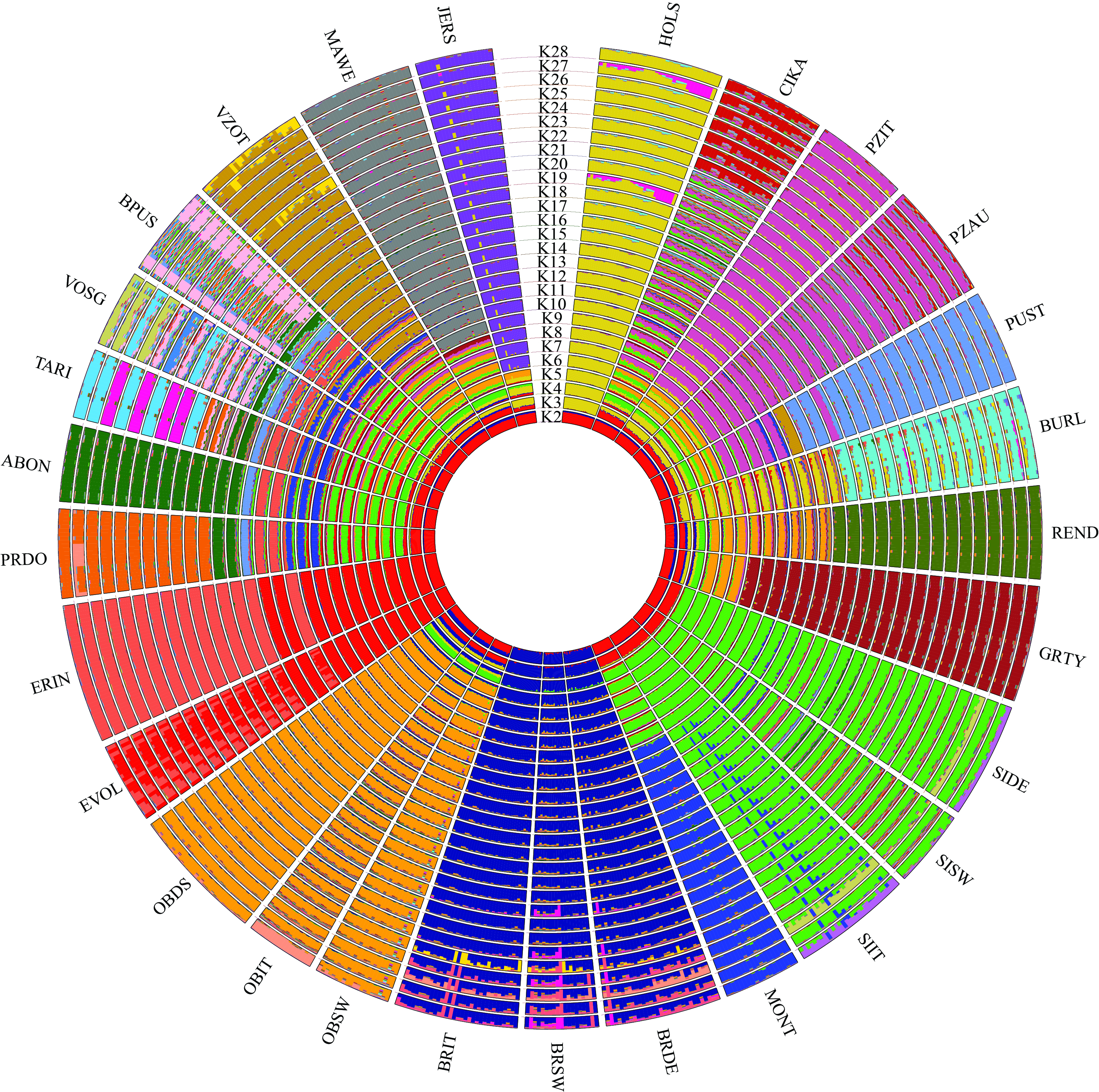

Supplement: Supplementary file 3 — Additional file 3: Figure S2. Admixture analysis plot in a circular fashion with all values of K (number of clusters) ranging from 2 to 28. [file 12711_2020_559_MOESM3_ESM.jpg]

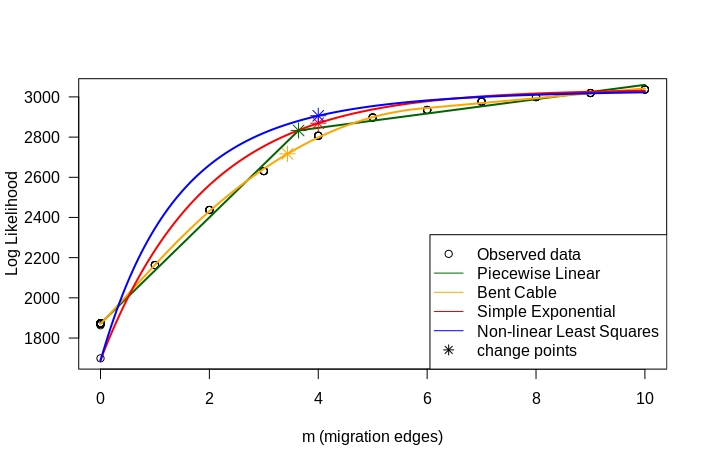

Supplement: Supplementary file 5 — Additional file 5: Figure S3. Increment in the log likelihood for all tested migration events calculated by using the optM function in the R package OptM. [file 12711_2020_559_MOESM5_ESM.jpeg]
