# Supplementary material for: The genetic heritage of Alpine local cattle breeds using genomic SNP data
Source: Genet Sel Evol. 2020 Jul 14;52:40. doi: 10.1186/s12711-020-00559-1 (PMC7362560; doi:10.1186/s12711-020-00559-1)
Supplement: Supplementary file 4 — Additional file 4: Table S2. Fixation indices (FST) between all pairs of breed populations analyzed in this study. [file 12711_2020_559_MOESM4_ESM.pdf]

	ERIN	EVOL	ABON	BURL	BPUS	BRDE	OBSW	BRSW	OBIT	CIKA	JERS	SIDE	BRIT	HOLS	SIIT	MONT	MAWE	OBDS	PZIT	PUST	PRDO	PZAU	REND	SISW	TARI	GRTY	VZOT	VOSG	
ERIN	0																											
EVOL	0.048	0																										
ABON	0.078	0.098	0																									
BURL	0.088	0.106	0.090	0																								0-0.025
BPUS	0.057	0.077	0.050	0.061	0																							0.026-0.050
BRDE	0.114	0.135	0.113	0.112	0.091	0																						0.051-0.100
OBSW	0.079	0.100	0.076	0.077	0.053	0.081	0																					0.101-0.150
BRSW	0.114	0.137	0.114	0.113	0.092	0.001	0.084	0																				0.151-0.200
OBIT	0.084	0.104	0.081	0.082	0.059	0.084	0.033	0.085	0																			
CIKA	0.060	0.079	0.057	0.061	0.036	0.089	0.051	0.088	0.056	0																		
JERS	0.156	0.175	0.158	0.143	0.132	0.179	0.147	0.182	0.151	0.126	0																	
SIDE	0.071	0.091	0.055	0.085	0.042	0.105	0.068	0.105	0.073	0.045	0.151	0																
BRIT	0.127	0.149	0.127	0.126	0.105	0.007	0.096	0.006	0.099	0.102	0.193	0.118	0															
HOLS	0.127	0.145	0.126	0.066	0.096	0.146	0.114	0.148	0.119	0.095	0.168	0.121	0.159	0														
SIIT	0.072	0.092	0.057	0.081	0.044	0.105	0.069	0.106	0.074	0.046	0.149	0.010	0.118	0.116	0													
MONT	0.091	0.112	0.080	0.099	0.064	0.125	0.089	0.126	0.094	0.068	0.169	0.065	0.138	0.134	0.057	0												
MAWE	0.089	0.108	0.085	0.087	0.064	0.106	0.071	0.107	0.076	0.061	0.152	0.077	0.119	0.121	0.078	0.097	0											
OBDS	0.082	0.101	0.079	0.082	0.058	0.081	0.015	0.084	0.033	0.055	0.146	0.072	0.096	0.118	0.073	0.092	0.074	0										
PZIT	0.089	0.110	0.088	0.070	0.062	0.112	0.076	0.112	0.082	0.046	0.146	0.084	0.126	0.089	0.082	0.099	0.085	0.081	0									
PUST	0.092	0.113	0.090	0.090	0.064	0.119	0.084	0.119	0.088	0.057	0.158	0.083	0.132	0.125	0.083	0.103	0.091	0.086	0.072	0								
PRDO	0.066	0.086	0.060	0.085	0.045	0.107	0.070	0.107	0.076	0.052	0.152	0.049	0.121	0.123	0.052	0.074	0.080	0.074	0.084	0.084	0							
PZAU	0.075	0.094	0.074	0.069	0.050	0.099	0.063	0.099	0.068	0.030	0.134	0.068	0.113	0.100	0.067	0.085	0.071	0.068	0.018	0.055	0.069	0						
REND	0.088	0.108	0.087	0.087	0.065	0.099	0.060	0.100	0.064	0.062	0.155	0.080	0.114	0.121	0.080	0.099	0.082	0.061	0.085	0.092	0.080	0.072	0					
SISW	0.070	0.091	0.055	0.083	0.042	0.104	0.066	0.105	0.071	0.043	0.153	0.015	0.118	0.120	0.022	0.065	0.076	0.070	0.082	0.081	0.048	0.066	0.078	0				
TARI	0.073	0.093	0.067	0.085	0.048	0.110	0.073	0.111	0.077	0.055	0.153	0.060	0.124	0.123	0.062	0.083	0.080	0.076	0.086	0.087	0.060	0.070	0.083	0.060	0			
GRTY	0.078	0.098	0.077	0.079	0.055	0.096	0.058	0.097	0.063	0.052	0.146	0.069	0.110	0.115	0.071	0.089	0.072	0.060	0.078	0.084	0.070	0.064	0.070	0.068	0.073	0		
VZOT	0.076	0.095	0.072	0.071	0.051	0.095	0.057	0.095	0.062	0.047	0.136	0.065	0.109	0.105	0.065	0.084	0.071	0.060	0.071	0.077	0.067	0.057	0.069	0.063	0.068	0.062	0	
VOSG	0.065	0.087	0.059	0.067	0.031	0.098	0.061	0.098	0.066	0.043	0.141	0.050	0.111	0.105	0.050	0.070	0.071	0.064	0.070	0.067	0.053	0.057	0.072	0.049	0.057	0.062	0.057	0	
